# Anlotinib induced type 1 diabetes: a case report

**DOI:** 10.3389/fonc.2025.1508645

**Published:** 2025-04-02

**Authors:** Jing Chen, Daohan Xia, Linqiu Ke

**Affiliations:** General Medicine Department, Chongqing General Hospital, Chongqing, China

**Keywords:** anlotinib, type 1 diabetes, TKI - tyrosine kinase inhibitor, VEGF - vascular endothelial growth factor, autoimmune disease

## Abstract

Studies have shown some tyrosine kinase inhibitors (TKIs) can influence glucose metabolism leading to either hypoglycemia or hyperglycemia which is reversable in most patients after treatment cessation. Anlotinib is a novel oral multi-target tyrosine kinase inhibitor (TKI) which has been approved for non-small cell lung cancer in China. Previous studies of anlotinib did not report it has any side effect on blood glucose, and there has been no case reporting type 1 diabetes associated with any TKI. The present case study, to our knowledge, was the first to report on an 81-year-old man with lung cancer who developed type 1 diabetes following 14 cycles treatment with TKI. The fasting plasma blood glucose and hemoglobinA1c (HbA1c) was 24.3mmol/L and 9.0%, respectively, and GADA (glutamic acid decarboxylase antibody) was more than 2000IU/ml (normal range is less than 10IU/ml) when he was diagnosed. We also conducted a literature review to explore the potential mechanism of anlotinib in inducing type 1 diabetes and recommend that self-monitoring blood glucose (SMBG) for fasting and random postprandial blood glucose at least once a week is needed for early identification of glucose dysregulation when using TKI drugs, and monthly fasting and random postprandial plasma glucose monitoring and HbA1c test every 3 months is also recommended if the SMBG protocol cannot be completed.

## Introduction

Tyrosine kinase inhibitors (TKIs) have been at the forefront in targeted chemotherapy for cancers over the past two decades, and anlotinib is a novel oral multi-target tyrosine kinase inhibitor (TKI) which has been approved for non-small cell lung cancer in China (1, 2). Studies have shown some TKIs can influence glucose metabolism leading to either hypoglycemia or hyperglycemia, which is reversable in most patients after treatment cessation (3). TKIs such as imatinib, erlotinib, and sunitinib can improve blood glucose concentration potentially in part due to preservation of functional β cell mass and increasement of insulin sensitivity or insulin secretion. While others such as Nilotinib, ceritinib and rociletinib may raise blood glucose levels attributing to pancreatic β-cell insulin secretion impairment or development of insulin resistance and inhibition of the insulin receptor (4, 5). However, the exact mechanism by which TKIs elicit an increase or a decrease in patients largely remains unknown. TKIs with similar structure and same target such as imatinib and nilotinib have shown opposite effects on glucose metabolism, even certain TKI such as imatinib has exhibited contrasting effects on blood glucose levels when used to treat different tumors (6, 7). Imatinib has shown glucose-lowing effects in the treatment of chronic myeloid leukemia, while in gastrointestinal stromal tumors, hyperglycemia has been reported with the use of imatinib in 0.1-1% of cases (8). In summary, TKIs exert markedly different effects on glucose metabolism depending on the specific TKI and the type of tumor being treated. Previous studies of anlotinib did not report it has any side effect on blood glucose level, and there has been no case reporting type 1 diabetes associated with any TKI, therefore, this case study is the first, to our knowledge, to report on a patient who developed type 1 diabetes following treatment with TKI.

## Case presentation

**Figure 1:**
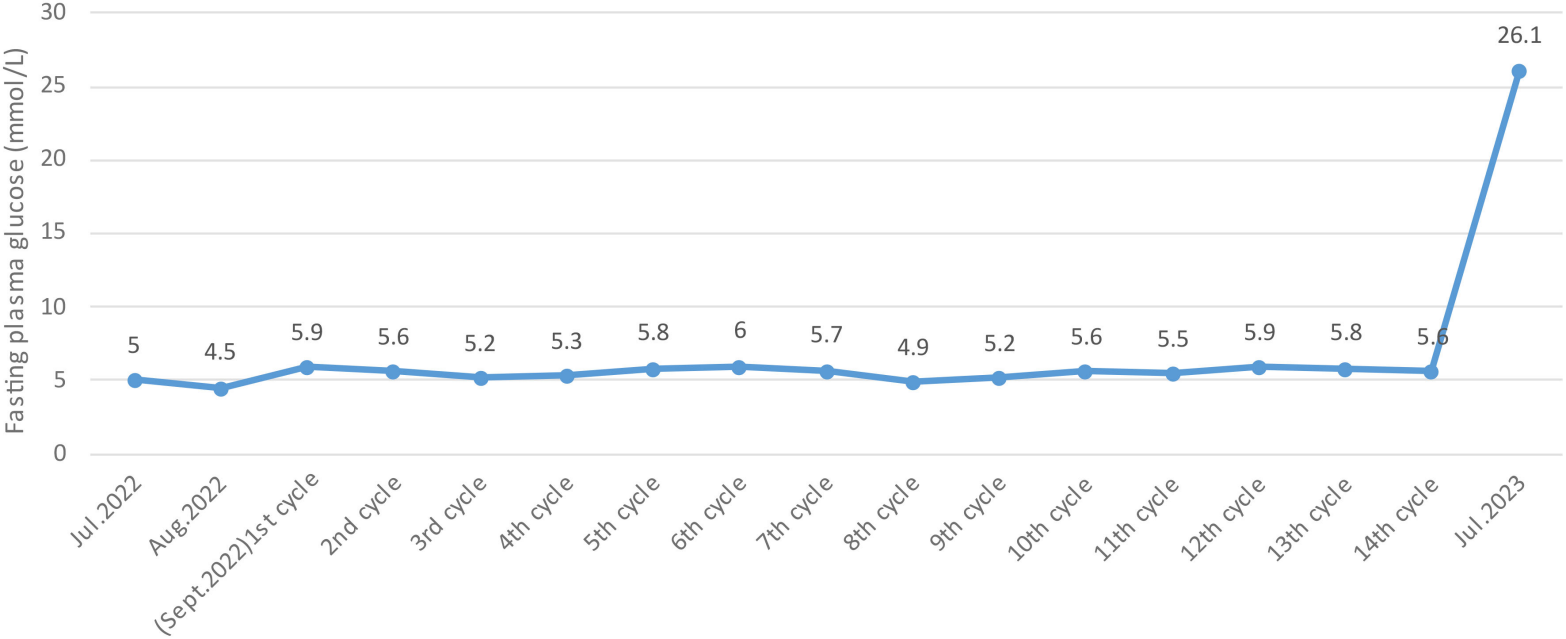
Fasting plasma glucose (before admission).

**Figure 2:**
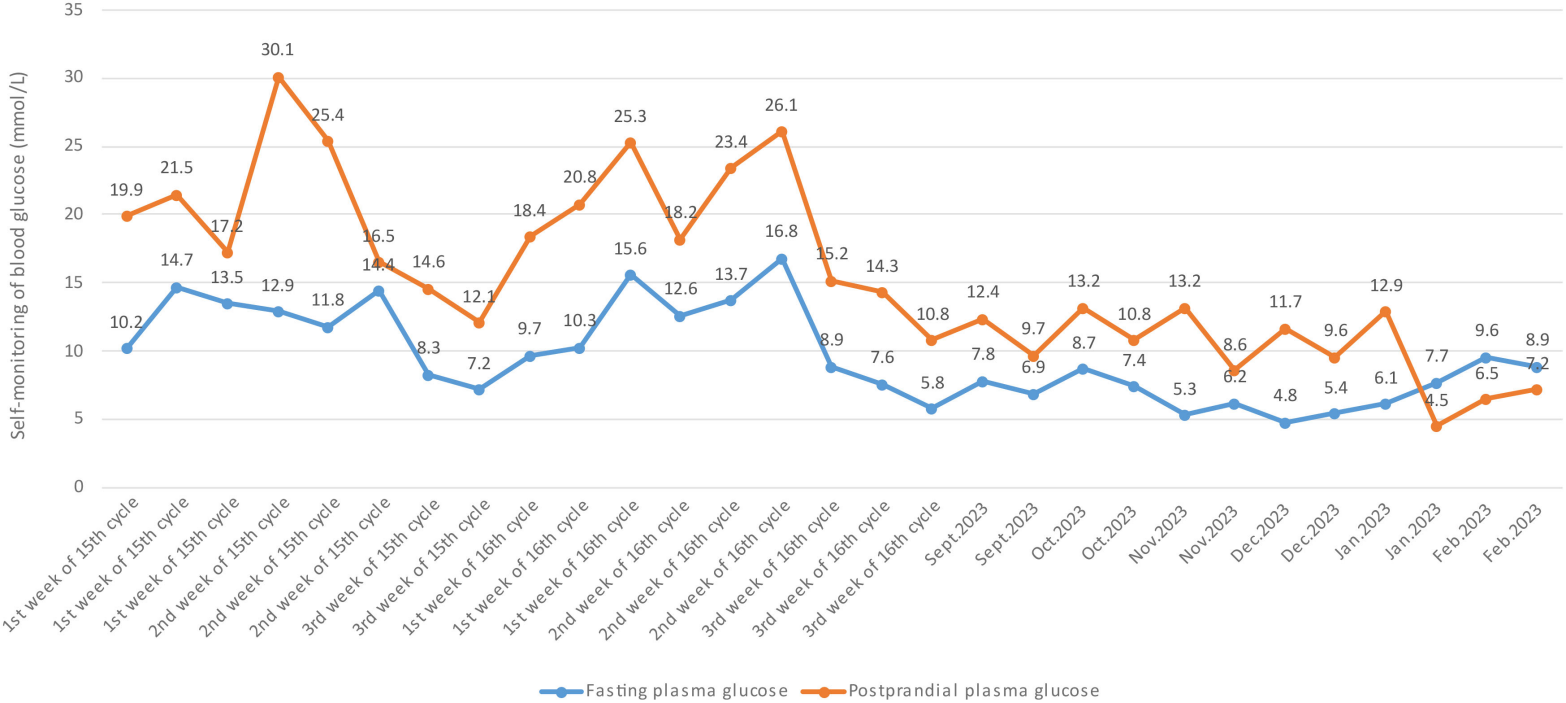
Fasting and random postprandial plasma glucose (after discharge).

An 81-year-old man without family history of diabetes or any chronic disease was diagnosed with lung adenocarcinoma (T4N2M1) in October 2020. He received gyroknife radiotherapy for left and right lung malignancies 12 times in November 2020, and 12 times for right lung malignancies in June 2021. In September 2022, the patient was administrated anlotinib 8mg orally once daily for 14 days every 3 weeks due to tumor progression. After 14 cycles treatment of anlotinib, his fasting plasma glucose was found at 26.1mmol/L and urine ketone was (+++) on July 12th 2023, then he was admitted to Endocrinology Department of Chongqing General Hospital (the blood glucose levels prior to anlotib administration and during the 14 cycles is shown in **Figure 1**). Other long term drug use history, anti-tumor drugs, medications known to potentially cause hyperglycemia such as steroids, records of SARS-CoV-2 vaccination or confirmed SARS-CoV-2 infection was not identified before admission. We only found history of short-term use of proton pump Inhibitors and anti-biotics before the onset of diabetes. Additionally, there were no symptoms of respiratory or gastrointestinal infections noted within one month before admission. Laboratory parameters were measured upon admission are detailed in **Table 1**. The patient was diagnosed with type 1 diabetes and was treated with insulin degludec in combination with insulin aspartate, and he was discharged on July 19th, 2023 with the hypoglycemic regimen (insulin degludec 5 units in the morning and insulin aspartate 4 units three times a day). The patient reported a significant increase in blood glucose levels after re-starting anlotinib one week after discharge. The highest blood glucose level recorded was 30.1 mmol/L and insulin dose was increased to maintain his glucose levels. Conversely, the blood glucose decreased significantly and the dosage of injected insulin was decreased after 14 days of anlotinib treatment (blood glucose level in the 15th and 16th cycle is shown in ure2). After two cycles of anlotnib treatment, his oncologist decided to discontinue the use of anlotinib due to tumor progression and its significant negative impact on glucose metabolism (the laboratory parameters after 16^th^ cycle is shown in **Table 1**). The patient had tried afatinib, erlotinib and osimertinib before he passed way in March 1^st^, 2024. The follow-up blood glucose level did not reveal any significant fluctuation before his death (the follow-up blood glucose level is shown in **Figure 2**). Informed consent had been obtained from the patient to present all his clinical data.

**Table 1:** Laboratory parameters on admission to Endocrinology Department of Chongqing General Hospital and after the 16^th^ cycle of anlotinib treatment.

Parameter	Value		Normal range
	on admission to Endocrinology Department of Chongqing General Hospital	after the 16th cycle of anlotinib treatment	
GADA (IU/ml)	> 2000	–	< 10
ICA (COI)	43.4	–	< 1
IAA (CDI)	< 1	–	< 1
Fasting blood glucose (mmol/L)	24.3	8.7	3.9-6.1
Fasting insulin (uIU/L)	0.83	–	4.03-23.5
FastingC-peptide (ng/ml)	0.13	–	0.3-3.73
Hemoglobin A1c, HbA1c(%)	9.0	8.1	4.5-6.3
Hemoglobin (g/L)	96	113	130.00-175.00
Serum albumin (g/L)	27.2	30.4	40-55
TC (mmol/L)	4.17	4.39	<5.20
TG (mmol/L)	0.67	0.58	0.00-1.70
LDL-C (mmol/L)	2.43	2.15	<3.37
Crea (umol/L)	87.7	90.1	57.0-111.0
AST (U/L)	16	17.8	15.0-40.0
ALT (U/L)	11.6	12.3	9.0-50.0
TSH (mIU/L)	5.32	–	0.3-5.5
FT3 (pmol/L)	4.41	–	3.08-7.00
FT4 (pmol/L)	18.0	–	11.57-22.36
pancreatic amylase (U/L)	63.6	–	35.0-135.0
lipase (U/L)	14.5	–	8.0-53.0

GADA, glutamic acid decarboxylase antibody; ICA, insular cellular antibody; IAA, insulin autoimmune antibody; TC, total cholesterol; LDL-C, low-density lipoprotein cholesterol; TG, triglyceride; AST, aspartate transaminase; ALT, alanine aminotransferase; TSH; thyroid stimulating hormone; FT3, free triiodothyronine; FT4, free thyroxine

## Discussion

With the development and application of new generation of TKI drugs such as anlotinib with more targets, their influence on glucose metabolism is more complex and harder to predict, some previously unknown forms of type 1 diabetes may occur when using new generation of TKI drugs. We conducted a literature review to explore the potential mechanism of anlotinib in inducing type 1 diabetes in this case. Firstly, Anlotinib exerts significant inhibitory effects on angiogenesis both *in vivo* and *in vitro* (9–11). The impact on islet blood vessels presents a dual effect on type 1 diabetes development. While it can impede the proliferation of islet blood vessels, thereby reducing inflammatory cell migration and restoring normal blood glucose metabolism (11, 12). It can also exacerbate islet capillary degeneration which may lead to islet hypoperfusion and subsequent abnormal hormone secretion from islet cells (13, 14). The mean progression-free survival (PFS) of anlotinib in lung adenocarcinoma was 5.5 months (15), however the patient had received anlotinib treatment for nearly 10 months before hyperglycemia was noticed in our case. Consequently, it is hypothesized that the potential harm resulting from prolonged anlotinib use, which excessively inhibits normal islet capillaries, outweighs the benefits of blocking increased blood supply, ultimately leading to abnormal insulin secretion and diabetes development. Secondly, Type 1 diabetes is an autoimmune disease caused by the immune-mediated destruction of insulin-producing pancreatic β cells (16). It is hypothesized that the neoantigen released by the tumor might be similar with Glutamic Acid Decarboxylase 65(GAD65) expressed by panreatic β cells in this case, therefore, activated T cell induced by the neoantigen might also destruct β cells through molecular mimicry. Anlotinib treatment can break immune tolerance of tumor microenvironment and inhibit tumor growth by enhancing CD8+ T cell infiltration which is dependent from the anti-angiogenesis effect (17). The enhanced anti-tumor immune response might also trigger the increased auto-immune attack to pancreatic β cells Which accelerated β cell reserve depletion, resulting the late-onset of type1 diabetes of this patient, it might also explain the phenomenon of blood glucose fluctuation during the last 2 cycles of anlotinib treatment (18, 19). Besides, recent evidence suggests anlotinib’s immunomodulatory effects may paradoxically amplify pre-existing autoimmunity. therefore, its capacity to enhance CD8+ T-cell infiltration could theoretically facilitate islet-directed immune attacks if the patient in our case was genetically predisposed (20). Thirdly, TKIs have been shown to have direct toxic effects on various organs and systems in the body including the pancreas. The toxicity of TKIs is attributed to their active metabolites, which are generated when TKIs interact with metabolic enzymes in the human body (5, 21, 22). It is hypothesized that the direct toxic effects of TKIs active metabolites on islet beta cells may play a role in the development of type 1 diabetes in this case. Future studies could leverage zebrafish xenotransplant models which successfully employed in TKI efficacy evaluation to dissect tissue-specific toxicity profiles (23).Fourthly, as a multi-target TKI with potent VEGFR2/FGFR/PDGFR inhibition, anlotinib’s metabolic impacts may differ from single-target agents which may have a significant impact on 14 endogenous metabolic pathways including glucose metabolism. This interference can lead to fluctuations in endogenous metabolites, causing both increases and decreases that result in side effects (5, 24). It is postulated that prolonged disruption of these metabolic pathways may negatively affect glucose metabolism and potentially contribute to the onset of type 1 diabetes in our case (7, 8).

In conclusion, to the best of our knowledge, the present case study was the first to report on a case of type 1 diabetes induced by anlotinib. The patient’s oncologist failed to identify the blood glucose dysregulation early although he regularly monitored fasting plasma glucose. Therefore, we recommend fasting blood glucose and HbA1c test to fully evaluate blood glucose before using TKI drugs. If the blood glucose is abnormal, diabetes-related autoantibodies should be further tested. We also recommend that SMBG for fasting and random postprandial blood glucose at least once a week is needed for early identification of glucose dysregulation when using TKI drugs. If the SMBG protocol can not be completed, we recommend to monitor fasting and random postprandial plasma glucose at least once o month and HbA1c every 3 months at outpatient clinics (25). In addition, further research is needed to explore the correlation and underlying mechanisms between TKI drugs and glucose metabolism.

## Limitation

We did not do SARS-CoV-2 antibodies test at the onset of diabetes to completely exclude the possibility of asymptomatic virus infection which might lead to the development of diabetes. We did not do type 1 diabetes high-risk genes (e.g. human leukocyte antigen, HLA-DR4/DQ8) or related autoantibodies tests before anotinib treatment to know whether the patient was susceptible for ty1 diabetes, either. HbA1c was not tested before using anlotinib to exclude the possibility that post-prandial blood glucose had already met the diagnostic criteria of diabetes before treatment. Either Pancreatic imaging to assess islet inflammation or T cell receptor library analysis which might help us explore the mechanism of the type1 diabetes in this case was not done.

## Data Availability

The raw data supporting the conclusions of this article will be made available by the authors, without undue reservation.
